# Development and Validation of a Liquid Chromatography/Tandem Mass Spectrometry Method for the Quantification of the GLP-1 Analog Semaglutide in Rat Plasma, and Its Application in a Pharmacokinetic Study

**DOI:** 10.3390/pharmaceutics18070770

**Published:** 2026-06-24

**Authors:** Jong-Min Kim, Kyoung-Ah Kim, Na-Young Yu, Dae-Duk Kim, Jeong Yeon Kang, Seung-Ki Baek, Jin-Woo Park, Ji-Young Park

**Affiliations:** 1 Department of Clinical Pharmacology and Toxicology, Anam Hospital, Korea University College of Medicine, Seoul 02841, Republic of Korea; jmk157@korea.ac.kr (J.-M.K.); kakim920@kumc.or.kr (K.-A.K.); 2 Institute of Medical Information and Data Convergence, Korea University College of Medicine, Seoul 02841, Republic of Korea; 3 College of Pharmacy and Research Institute of Pharmaceutical Sciences, Seoul National University, Seoul 08826, Republic of Korea; nyyu8520@snu.ac.kr (N.-Y.Y.); ddkim@snu.ac.kr (D.-D.K.); 4 QuadMedicine R&D Centre, QuadMedicine Inc., Seongnam 13209, Republic of Korea; jkang@quadmedicine.com (J.Y.K.); bsk@quadmedicine.com (S.-K.B.); 5 Department of Neurology, Anam Hospital, Korea University College of Medicine, Seoul 02841, Republic of Korea

**Keywords:** semaglutide, GLP-1, LC-MS/MS

## Abstract

**Background/Objectives:** Semaglutide, a long-acting glucagon-like peptide-1 (GLP-1) analog for type 2 diabetes and obesity, requires sensitive and high-throughput bioanalytical methods to support pharmacokinetic studies. However, previously reported liquid chromatography–tandem mass spectrometry (LC–MS/MS) assays have been limited by lengthy run times (~18 min) and suboptimal sensitivity. This study aimed to develop and validate a rapid, sensitive LC–MS/MS method for quantifying semaglutide in plasma. **Methods:** Plasma samples (50 μL) were prepared by acetone-mediated protein precipitation followed by solid-phase extraction. Chromatographic separation was performed on a Cadenza CD-C18 MF column within 9 min, using positive electrospray ionization in multiple reaction monitoring mode with the transitions *m*/*z* 1029.4 → 110.1 for semaglutide and *m*/*z* 938.9 → 109.9 for liraglutide (internal standard). Validation followed the U.S. Food and Drug Administration (FDA) bioanalytical guidelines. **Results:** The assay showed a lower limit of quantification of 1 ng/mL with linearity across 1–500 ng/mL (R^2^ = 0.9999), with sharp peak shape and no carryover. Intra- and inter-day accuracies were 95.69–103.76% and 94.93–100.08%, with precision ≤4.50% and ≤5.88%. Recovery (93.05–107.95%) and matrix effects (96.34–104.12%) were consistent across quality control levels, and the analyte was stable under all tested conditions. The method was successfully applied to a pharmacokinetic study in Sprague–Dawley rats following subcutaneous administration of 50 μg semaglutide. **Conclusions:** The validated method offers shorter analysis time, improved sensitivity, and reduced sample volume compared with previously reported assays, supporting its application in preclinical pharmacokinetic studies of semaglutide and related GLP-1 analogs.

## 1. Introduction

Semaglutide (Ozempic^®^, Novo Nordisk A/S, Bagsværd, Denmark), a once-weekly subcutaneous glucagon-like peptide-1 (GLP-1) analog for type 2 diabetes mellitus (T2DM) management [1,2], shares significant structural similarity with the first GLP-1 analog liraglutide approved for the treatment of obesity, exhibiting a 94% homology to the native human GLP-1 amino acid sequence [3]. When compared to the latter, semaglutide features critical modifications—amino acid substitutions at positions 8 (alanine to α-aminoisobutyric acid) and 34 (lysine to arginine), and acylation of lysine with a spacer and C-18 fatty diacid chain at position 26 [3,4]—that increase its resistance to dipeptidyl peptidase-4 (DPP-4) degradation and renal clearance [3,4]. These alterations significantly prolong its half-life from less than 2 min (for native GLP-1) to between 165–183 h [5,6], enhancing glycemic control, reduction in body weight, and providing cardiovascular benefits, as shown in the SUSTAIN trial [7,8,9,10,11,12].

Existing pharmacokinetic analyses of semaglutide [13,14,15,16], which are heavily dependent on LC–MS/MS methodologies, face challenges due to protracted run times [17] and limited quantification ranges [18], impacting both sample stability and cost-effectiveness. Despite recent expansions in the quantification range, the reported run times remain lengthy at approximately 18 min [17]. Recognizing the necessity for more efficient analyses, this study proposes a sophisticated LC–MS/MS technique designed for swift and precise quantification of semaglutide in plasma, thus addressing the shortcomings of previous methods.

Consequently, we developed an optimized LC–MS/MS method that offers a shorter run time, sharp peak shape, and no carryover compared with selected previously reported LC–MS/MS methods. This novel method considerably reduces the analysis duration, enhances sample stability, and reduces laboratory operational costs [19,20,21,22]. Furthermore, its increased throughput capacity allows for the processing of a higher volume of samples in a given period, providing critical advantages in settings where large-scale sample analysis is required, such as in preclinical pharmacokinetic studies. In addition, the low lower limit of quantification (LLOQ) of 1 ng/mL and improved peak resolution without peak tailing ensure more accurate results [23].

## 2. Materials and Methods

### 2.1. Reagents

**Figure 1:**
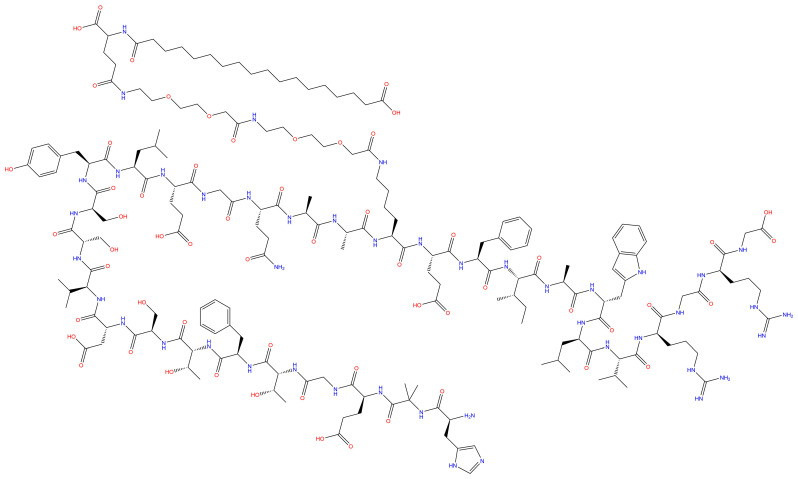
Chemical structure of semaglutide (C_187_H_291_N_45_O_59_; MW 4113.6 g/mol).

Semaglutide (>95%; Shenzhen JYMed Technology Co., Ltd., Shenzhen, China) was donated by QuadMedicine (Seongnam, Republic of Korea), and the internal standard (IS) liraglutide (>95%) was purchased from Toronto Research Chemicals Inc. (Toronto, ON, Canada). The chemical structure of semaglutide is shown in Figure 1. High Performance Liquid Chromatography (HPLC) grade formic acid was purchased from Sigma-Aldrich (St. Louis, MO, USA), acetonitrile and methanol were purchased from J. T. Baker, Inc. (Phillipsburg, NJ, USA), and acetone was obtained from Merck (Darmstadt, Germany). Ultrapure water was purified using a MembraPure water purification system (Bodenheim, Germany). Stock solutions of 1 mg/mL semaglutide and 1 mg/mL liraglutide (IS) were prepared by dissolving the molecules individually in dimethyl sulfoxide (DMSO, 99.9%, Sigma-Aldrich (St. Louis, MO, USA)). Rat plasma with K_2_ EDTA (Innovative Research, Novi, MI, USA) was used for the standard curve and quality control (QC) samples. All the other reagents used in this study were of high purity and analytical grade.

### 2.2. LC-MS/MS Instrumentation

Plasma samples spiked with semaglutide and the IS (liraglutide) were analyzed using an LC–MS/MS system consisting of a Nanospace series SI-2 HPLC system (Shiseido Co., Ltd., Tokyo, Japan) and an API-4000 tandem mass spectrometer (Applied Biosystems SCIEX, Framingham, MA, USA) equipped with an electrospray ionization source. The Nanospace series SI-2 HPLC system consisted of a column oven, binary pump, vacuum degasser, and autosampler and was controlled by Analyst ver. 1.5.1 software (Applied Biosystems).

### 2.3. Liquid Chromatography–Mass Spectrometry (LC–MS) Conditions

Chromatographic separation was carried out on a Cadenza CD-C18 MF column (150 mm × 2.0 mm, 3 μm (Imtakt, Kyoto, Japan)) coupled with a guard-column (Phenomenex, Torrance, CA, USA). The column oven temperature was set at 40 °C and gradient elution was performed using acetonitrile containing 0.2% formic acid (mobile phase A) and distilled water with 1% formic acid (mobile phase B). The total analysis time was 9 min. The detailed gradient elution conditions and flow rates are listed in Supplementary Table S1.

The mass spectrometer combined with an electrospray ionization source was operated in positive ion mode using multiple reaction monitoring (MRM). The selected mass transition ion pair for MRM analysis were *m*/*z* 1029.4 → 110.1 (M1) and *m*/*z* 1029.4 → 135.9 (M2) for semaglutide, and *m*/*z* 938.9 → 109.9 (M1) and *m*/*z* 938.9 → 136.0 (M2) for the IS. Other optimal mass spectrometer operating conditions were as follows: collision gas (CAD) 5 psi, curtain gas (CUR) 25 psi, ion source gas 1 (GS1) 55 psi, ion source gas 2 (GS2) 55 psi, ion spray voltage (IS) 5500 V, and ion source temperature (TEM) 550 °C. The collision energy (CE) and collision cell exit potential (CXP) were set to 121 and 6 V for semaglutide (M1) and 91 and 14 V for semaglutide (M2), respectively. In addition, those for the IS (M1 and M2) were set to 129 and 22 V, and 95 and 12 V, respectively. The declustering potential (DP) and entrance energy (EP) for both molecules were set to 76 and 10 V, respectively.

### 2.4. Calibration Standard and QC Samples

Stock solutions (1 mg/mL) of semaglutide and the IS were separately dissolved in DMSO, aliquoted, and stored at −80 °C until use. Each stock solution was serially diluted with 50% acetonitrile (with distilled water) (1:1, *v*/*v*) to prepare working solutions for calibration standards from 10 μg/mL to 0.02 μg/mL. Standard curves were prepared by spiking 10 μL of serially diluted semaglutide working solution in 190 μL of blank rat plasma and mixing well in a 1.5 mL polypropylene tube to obtain final concentrations of 1, 5, 10, 50, 100, 250, and 500 ng/mL. A blank plasma sample was prepared as described above, with the same proportion of DMSO (without semaglutide) mixed in the matrix. The percentage of DMSO in all prepared rat plasma samples was less than 0.05%.

QC samples were prepared at four different concentration levels: 1 ng/mL (LLOQ), 5 ng/mL (low), 100 ng/mL (medium), and 500 ng/mL (high). In accordance with the FDA bioanalytical method validation guidance, the calibration standards and QC samples were prepared independently from separately prepared stock solutions.

### 2.5. Sample Preparation

A 50 μL of rat plasma containing semaglutide was transferred into a 1.5 mL polypropylene tube and 10 μL of the IS solution (1 μg/mL) was added. The mixture was then vortexed with 100 μL of acetone for 5 min and then centrifuged at 15,000× *g* at room temperature for 15 min for protein precipitation. Subsequently, the supernatant was used for solid phase extraction (SPE) on a strata-X-A 30 mg/mL column cartridge (Phenomenex, Torrance, CA, USA).

The SPE procedure was as follows: The column was pre-conditioned with acetonitrile (1 mL), then equilibrated with distilled water (1 mL), and loaded with 140 μL of the precipitated sample supernatant. The cartridge was then washed with 25 mM ammonium acetate (1 mL) followed by acetonitrile (1 mL). Following this, it was eluted twice with 500 μL of acetonitrile containing 5% formic acid, and 700 μL of the eluent was transferred to a new tube and evaporated to dryness using a centrifugal vacuum evaporator at 25 °C for 1.5 h. Finally, the residue was reconstituted with 40 μL of reconstitution solution (mobile phase A: mobile phase B = 3:2, *v*/*v*), the sample was centrifuged, and the supernatant was transferred to a low protein binding tube, placed in an auto sampler, and analyzed by injecting 10 μL into the LC–MS/MS system.

### 2.6. Blood Sample Collection

The pharmacokinetics of semaglutide in rats was investigated using the developed and validated LC–MS/MS method. All animal experiments were performed according to approved Institutional Animal Care and Use Committee protocols of Seoul National University (Seoul, Republic of Korea; SNU-211213-7). Eight male Sprague–Dawley rats (230–260 g, male, 8-week-old) were purchased from Koatech Co. (Pyeongtaek, Republic of Korea). Blood samples were collected from the animals after subcutaneous injection of 50 μg semaglutide dissolved in phosphate-buffered saline. The samples were drawn immediately before injection (0 h) and 0.08, 0.17, 0.25, 0.5, 1, 2, 4, 8, and 24 h after administration into K_2_ EDTA tubes. The collected samples were centrifuged (3000× *g*, 4 °C) for 10 min and the isolated plasma samples were stored at −80 °C until analysis, and unfrozen plasma samples were centrifuged at 14,000× *g* for 5 min before use.

### 2.7. Method Validation

For method validation, selectivity, linearity, accuracy and precision, recovery, matrix effects, and stability experiments were carried out in line with U.S. Food and Drug Administration (FDA) guidelines [24].

#### 2.7.1. Selectivity and Specificity

Selectivity and specificity were evaluated using six independent rat plasma lots by comparing drug-free blank plasma with plasma spiked with semaglutide and IS. In the semaglutide spiked sample, it was determined whether the blank plasma exceeded 20% of the LLOQ, and in that for the IS, it was investigated whether the blank plasma exceeded 5% of the average response of the calibrator and QC sample. In addition, for each comparative verification, we confirmed whether exogenous or endogenous chemical components interfered with the analysis of semaglutide and the IS in the investigated concentration range.

#### 2.7.2. Linearity

Linearity was assessed using standard mixture samples containing the analytes at a concentration range of 1–500 ng/mL as mentioned previously in the Calibration standard and QC samples section. The slope, intercept, and determination coefficient of the calibration curve, consisting of analyte concentrations versus the peak area ratios of the analyte to the IS, were calculated using non-weighted linear regression analysis.

In accordance with FDA guidelines [24], the LLOQ was determined as the lowest standard of the calibration curve with accuracy (%bias) and precision (%CV) within ±20%.

#### 2.7.3. Accuracy and Precision

The accuracy and precision of the method was determined using four QC sample concentrations (LLOQ, low, medium, and high) (Section 2.4). The intraday accuracy and precision were determined by evaluating the QC sample on the same day, while for inter-day accuracy and precision, these were evaluated on three consecutive days. The accuracy and precision values of these experiments were determined as bias (%) and CV (%), respectively, and according to the FDA guidance approval criteria [24]. The bias and CV values were assessed to ensure that they met the ±15% criterion (except ±20% for the LLOQ) at each concentration level.

#### 2.7.4. Recovery and Matrix Effect

The recovery and matrix effects were determined by evaluating three QC samples (low, medium, and high) spiked with the analyte and IS. The matrix effect was evaluated at the same three QC concentrations using six independent rat plasma lots. Recovery was assessed by comparing the detection response of the sample to which the analyte was added after extracting the blank plasma (post-extracted sample) to that of the sample obtained by extracting the plasma after adding the analyte to the blank plasma (pre-extracted sample). The matrix effect was evaluated by comparing the post-extracted sample with the sample prepared in the absence of a biological matrix (unextracted sample).

The recovery and matrix effect were calculated as follows: Recovery (%) = peak area of (pre-extracted samples/post-extracted samples) × 100; Matrix effect (%) = (analyte/IS peak area ratio of post-extracted samples/analyte/IS peak area ratio of un-extracted samples) × 100, expressed as the IS-normalized matrix factor.

#### 2.7.5. Stability

Stability at different conditions or environments, viz. bench-top (room temperature for 8 h), auto-sampler (room temperature for 24 h), long-term (−80 °C for 5 months), and freeze/thaw (3 cycles) was confirmed using QC samples (low, medium, high). The accuracy (% nominal) at all conditions was assessed to ensure that it met the acceptance criteria of ±15% specified in the FDA guidelines [24].

#### 2.7.6. Dilution Integrity

Dilution integrity was evaluated to confirm that samples exceeding the ULOQ could be accurately quantified after dilution. QC samples were prepared at 1000 ng/mL, above the ULOQ (500 ng/mL), and diluted 2-fold with blank rat plasma to bring the concentration within the calibration range. Five replicates (*n* = 5) were analyzed, and the accuracy and precision were assessed against the ±15% acceptance criteria specified in the FDA guidelines [24].

#### 2.7.7. Carryover

Carryover was assessed by injecting a blank sample immediately after the ULOQ (500 ng/mL) sample, repeated in five replicates (*n* = 5). The autosampler needle was washed by sonication for 5 s between injections. In accordance with the ICH guidelines [25], the carryover in the blank sample was required not to exceed 20% of the LLOQ response for the analyte and 5% for the IS.

#### 2.7.8. Incurred Sample Reanalysis (ISR)

Incurred sample reanalysis (ISR) complied with the FDA guidelines [24] and ICH guidelines on bioanalytical method validation [25] to demonstrate the reproducibility of the method. The pharmacokinetic samples used were selected at two different concentrations (around C_max_, the elimination phase). ISR was performed within the stability period of the analyte on different days. It was evaluated as the difference (%) between the repeat value and the initial value (repeat value − initial value/mean value ×100) and had to meet the ±20% criterion.

### 2.8. Pharmacokinetic Study

The pharmacokinetic parameters of semaglutide were determined by non-compartmental analysis using Phoenix^®^ WinNonlin^®^ software (ver. 8.1, Certara^TM^, Princeton, NJ, USA). The maximum peak plasma concentration (C_max_) and time to reach C_max_ (T_max_) were assessed from the observed plasma concentration-time data. The total area under the time-plasma concentration curve (AUC_last_) was calculated over the dosing interval of 24 h using the linear trapezoidal rule. The AUC from 0 h to infinity (AUC_inf_) was calculated using the curvilinear area under the curve obtained by extrapolating the time of dosing to infinity based on the last observed (OBS) concentration. The half-life (t_1/2_) was calculated as t_1/2_ = ln(2)/Ke, and the apparent clearance (CL/F), the volume of plasma cleared of drug per unit time, was calculated as CL/F = Dose/AUC_inf_.

## 3. Results

### 3.1. Selectivity and Specificity

**Figure 2:**
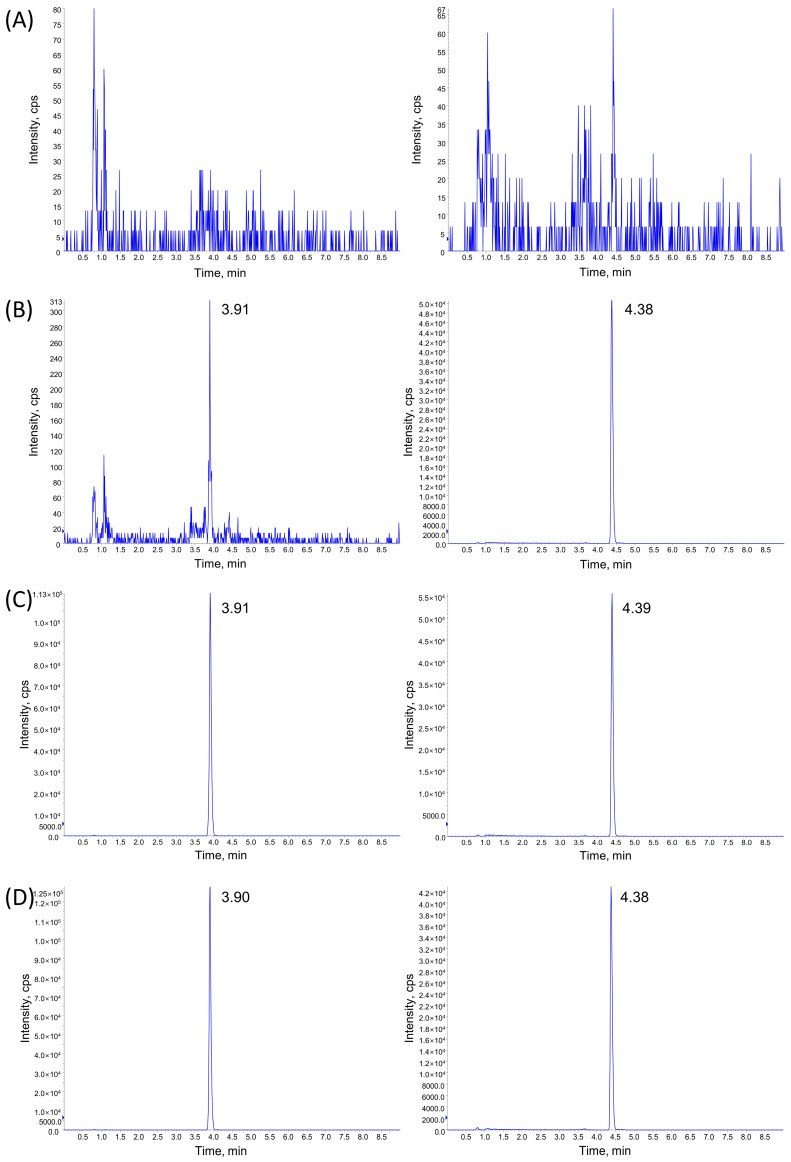
Representative MRM chromatograms of semaglutide (*m*/*z* 1029.4 → 110.1, left column) and IS (*m*/*z* 938.9 → 109.9, right column). (**A**) Blank plasma; (**B**) plasma spiked at the LLOQ (1 ng/mL); (**C**) plasma spiked at the ULOQ (500 ng/mL); (**D**) plasma sample obtained 8 h after subcutaneous administration of 50 μg semaglutide.

The total ion chromatograms of semaglutide and the IS were monitored using six independent blank rat plasma lots, as shown in Figure 2. There was no internal or external interference at the retention times consistent with the semaglutide and IS peaks in any of the lots examined, with both peaks constantly appearing at the same retention time, indicating the selectivity and specificity of the method.

### 3.2. Linearity and LLOQ

The LLOQ of the method was determined as the lowest concentration on the calibration curve that could be quantified with accuracy and precision within ±20% according to FDA guidelines [24]. The prepared LLOQ samples were analyzed five times separately from those of the calibration curve. The LLOQ for semaglutide was 1 ng/mL. This was 3-fold lower than that measured in human plasma samples in previous reports [18], indicating a considerable improvement in sensitivity.

The nominal concentration range of semaglutide was 1–500 ng/mL, and the area under the peak ratio was proportional to the analyte concentration. The standard curve was linear over the concentration range and was described by a least-squares line. The equation of the line was Y = 0.004541 × X − 0.00005728, the coefficient of determination (R^2^) was 0.9999, and no additional weighting factor was included (Supplementary Figure S1). The FDA and ICH guidelines recommend applying the simplest model that adequately describes the concentration–response relationship, together with appropriate weighting and goodness-of-fit assessment. The non-weighted model met the acceptance criteria at all calibration levels, including the lowest concentrations (Table 1), where the choice of regression weighting most strongly affects performance. Furthermore, the back-calculated concentrations of the calibration standards were within the acceptance criteria at all levels (Supplementary Table S2). The non-weighted model therefore constituted the simplest model adequately describing the relationship, and no weighting factor was required.

### 3.3. Accuracy and Precision

The intra- and inter-day accuracy and precision values of the semaglutide method are presented in Table 1. Intra- and inter-day values were determined using four different QC sample concentrations (LLOQ, low, medium, and high). Five QC samples were analyzed three times using the calibration curve. The intra- and inter-day accuracy results were 95.69–103.76% and 94.93–100.08%, respectively, and the precision values were ≤4.50% and ≤5.88%, respectively, which satisfied the criteria set by the FDA guidelines [24].

### 3.4. Recovery and Matrix Effect

The mean extraction recovery and matrix effects of the semaglutide method were evaluated for three different QC sample concentrations (low, medium, and high), as indicated in Table 2. The mean recovery values were 93.05–107.95%, the matrix effect was 96.34–104.12%, and the variability (%CV) of the mean recovery and matrix effect were 1.63–2.47% and 2.96–9.84%, respectively. Considering that the mean recovery and matrix effects of semaglutide were consistent and reproducible, the suitability of the sample preparation method was confirmed.

### 3.5. Stability

Stability analysis was carried out at four different conditions (bench-top, long-term, auto-sampler, and freeze/thaw cycles), and the results are presented in Table 3. These experiments were performed on three QC sample concentrations (low, medium, high), and the accuracy (% nominal) for each test was 99.42–101.39%, 95.68–100.29%, 100.06–101.09%, and 93.01–101.56%, respectively. Further, the variability (%CV) was 1.46–2.53%, 0.68–2.01%, 0.54–8.05%, and 2.32–4.65%, respectively. All the aforementioned results were in accordance with the acceptance criteria set in the FDA guidelines [24].

### 3.6. Dilution Integrity

The 2-fold diluted QC samples (1000 ng/mL) showed an accuracy of 101.32% and a precision (CV) of 2.24%, both within the acceptance criteria specified in the FDA guidelines [24], confirming that samples with concentrations above the ULOQ could be reliably quantified after dilution.

### 3.7. Carryover

The blank samples injected after the ULOQ showed a carryover of 5.3% of the LLOQ response for the analyte and 0.5% for the IS, both within the acceptance criteria specified in the ICH guidelines, confirming the absence of significant carryover.

### 3.8. ISR

The results of the ISR tests are presented in Table 4. For all 16 samples analyzed, the calculated difference values were within ±20% (−7.59–9.06%), which satisfied the acceptance criteria for bioanalytical method validation [25], indicating that the method was consistent and had acceptable reproducibility.

### 3.9. Pharmacokinetic Study

**Figure 3:**
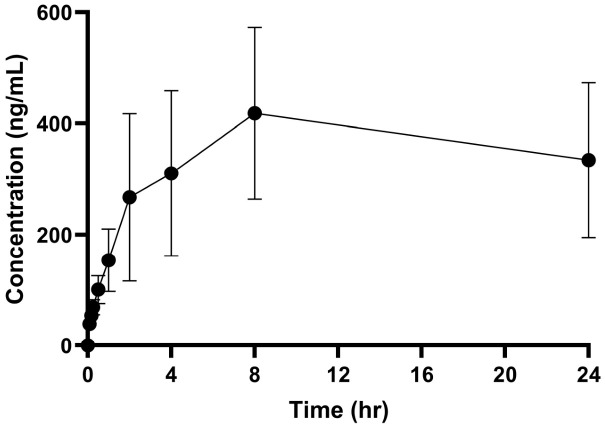
Mean plasma concentration–time profile of semaglutide following a single subcutaneous injection of 50 μg in male Sprague–Dawley rats (*n* = 8). Data are presented as mean ± SD.

The developed and validated method was used to measure the plasma concentration and pharmacokinetic parameters of semaglutide in eight Sprague–Dawley rats. Plasma samples with concentrations exceeding the ULOQ were diluted 2-fold with blank rat plasma and reanalyzed. The mean plasma concentration-time profile after administration of 50 μg of semaglutide by subcutaneous injection is depicted in Figure 3. Pharmacokinetic parameters are listed in Table 5. The systemic exposure of semaglutide was determined after subcutaneous injection of 50 μg semaglutide, and a maximum peak concentration (C_max_) of 419.07 ± 155.95 ng/mL was reached at a mean T_max_ of 7.25 ± 2.12 h. The exposure parameters C_max_, T_max_, and AUC_last_ were determined directly from the measured concentration–time profile. The terminal-phase parameters, including the apparent half-life (t_1/2_, 61.03 ± 30.88 h), AUC_inf_, and apparent clearance (CL/F, 0.002 ± 0.002 L/h), were derived in part through extrapolation beyond the final sampling point and are presented as preliminary estimates.

## 4. Discussion

**Figure 4:**
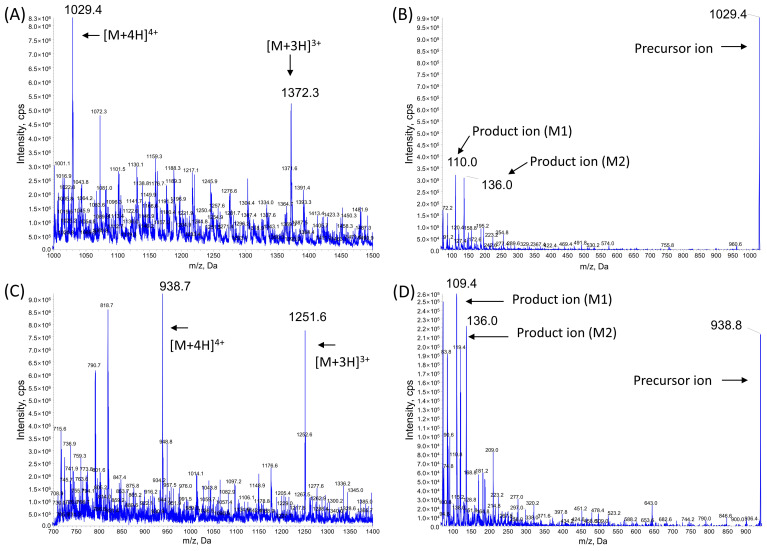
Mass spectra of semaglutide and liraglutide (IS) acquired in positive electrospray ionization mode. (**A**) Q1 full-scan spectrum of semaglutide; (**B**) product ion spectrum of semaglutide; (**C**) Q1 full-scan spectrum of liraglutide (IS); (**D**) product ion spectrum of liraglutide (IS).

Semaglutide has a very large molecular weight of 4113.6 g/mol. Figure 4A shows the Q1 full-scan mass spectrum of semaglutide obtained from an API-4000 tandem mass spectrometer, in which multiple-charged ions with protonated semaglutide were observed. The most abundant precursor ion in the Q1 full scan mass spectrum was *m*/*z* 1029.4 of [M+4H]^4+^, from which we performed the most optimized MRM transition, as shown in Figure 4B, and selected the most abundant and sensitive product ions, *m*/*z* 1029.4 → 110.1 (M1) and *m*/*z* 1029.4 → 135.9 (M2), through several reanalyses. Similarly, for liraglutide, MRM transitions from the most abundant precursor ions to the most sensitive product ions were selected in the same manner, with *m*/*z* 938.9 → 109.9 (M1) and *m*/*z* 938.9 → 136.0 (M2) being selected (Figure 4C,D).

**Figure 5:**
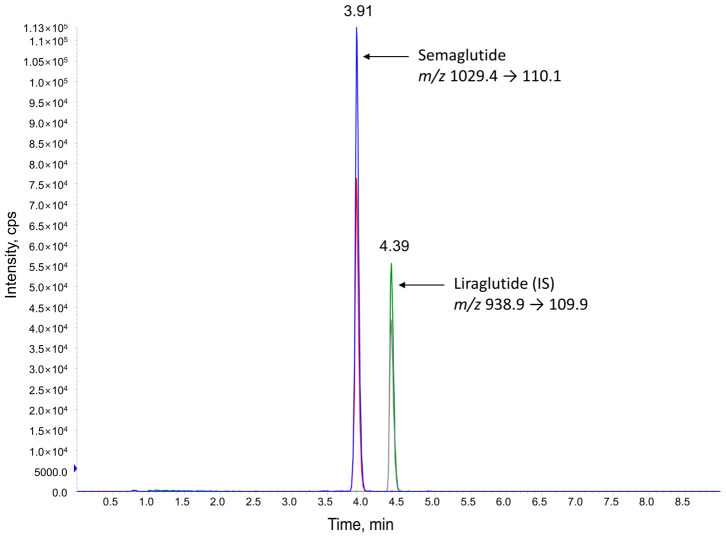
Representative LC–MS/MS MRM chromatogram of a rat plasma sample spiked with semaglutide and IS, showing baseline-resolved peaks at retention times of 3.91 min (semaglutide) and 4.39 min (IS). Each analyte is monitored by a quantifier (M1) and a qualifier (M2) transition: semaglutide M1 (blue, *m*/*z* 1029.4 → 110.1) and M2 (red, *m*/*z* 1029.4 → 135.9), and liraglutide (IS) M1 (green, *m*/*z* 938.9 → 109.9) and M2 (gray, *m*/*z* 938.9 → 136.0).

Chromatographic conditions were optimized to achieve the highest peak resolution of semaglutide with minimal matrix interference by examining mobile phase composition and analytical column. Regarding the mobile phase, acetonitrile was superior to methanol as the organic solvent, and the inclusion of formic acid enhanced the peak intensity of semaglutide; therefore, 0.2% and 1% formic acid were added to acetonitrile and distilled water, respectively. The Cadenza CD-C18 MF column (150 mm × 2.0 mm, 3 μm) was selected for chromatographic separation. Peptides such as semaglutide can interact with stainless steel column hardware, potentially causing peak tailing; thus, a metal-free polyetheretherketone (PEEK) column was used to mitigate such interactions, yielding sharp and symmetrical peaks for both semaglutide and the IS (Figure 5). With an optimized gradient elution profile (Supplementary Table S1), semaglutide and the IS showed consistent retention times of 3.91 and 4.39 min, respectively, with no observable carryover.

In the initial step of sample preparation, protein precipitation with acetonitrile and methanol was evaluated. Acetonitrile yielded suboptimal recovery, while methanol resulted in high background noise and considerable baseline interference. In contrast, high recovery and low noise were observed when using acetone as a precipitation solvent; additionally, for further enhancement, solid-phase extraction (SPE) using an anion-exchange cartridge was applied, with the wash and elution steps optimized to further reduce noise and improve recovery. This resulted in a recovery of 93.05–107.95%.

The present method offers two key advantages over previously reported LC–MS/MS assays for semaglutide. First, the chromatographic run time was reduced from approximately 18 min in a previously reported method [17] to 9 min, representing a 50% reduction in analysis time and enabling higher sample throughput. Second, the LLOQ of 1 ng/mL was 3-fold lower than the 3 ng/mL previously reported [18], providing improved sensitivity for detecting low semaglutide concentrations during the early absorption phase and the terminal elimination phase. Together with the small plasma sample volume requirement (50 μL), these improvements support the suitability of the present method for preclinical pharmacokinetic studies.

The validated method was applied to characterize the plasma pharmacokinetics of semaglutide in rats following subcutaneous administration. Semaglutide reached its peak plasma concentration at a mean T_max_ of 7.25 h, reflecting a relatively slow subcutaneous absorption. The low LLOQ (1 ng/mL) and the dense early sampling schedule (from 0.08 h) enabled the absorption phase to be delineated in detail, and the directly observed exposure parameters (C_max_, T_max_, and AUC_last_) were reliably quantified throughout the sampled interval. These results confirm that the method possesses sufficient sensitivity and reliability to support pharmacokinetic profiling of semaglutide in preclinical studies.

With respect to the in vivo application, this study has one limitation. As a long-acting, acylated GLP-1 analog, semaglutide exhibits a prolonged terminal half-life, and the 24-h blood sampling window did not fully capture its terminal elimination phase. The terminal-phase parameters (t_1/2_, AUC_inf_, and CL/F) were therefore derived in part through extrapolation and are presented as preliminary estimates rather than a definitive pharmacokinetic characterization. However, as the objective of the in vivo experiment was to demonstrate the applicability of the validated method to authentic biological samples rather than to provide a complete disposition profile, the reliably quantified exposure parameters (C_max_, T_max_, and AUC_last_) within the sampled interval are sufficient for this purpose. A complete characterization of the terminal disposition of semaglutide will require a dedicated study employing an extended sampling schedule beyond 24 h, for which the validated method provides a suitable analytical basis.

## 5. Conclusions

We developed and validated a novel, sensitive, and rapid LC–MS/MS method for quantifying semaglutide, a peptide used as an antidiabetic and anti-obesity medication, in rat plasma. The chromatographic run time was 9 min. This method can easily be put to practical use in routine laboratories for analyses using only a small amount of plasma. The method was validated in biological samples with satisfactory results, and its suitability for application in pharmacokinetic studies was successfully demonstrated, allowing the quantification of blood levels of semaglutide.

## Figures and Tables

**Table 1:** Intra-and inter-day assessments of accuracy and precision for semaglutide using QC samples (*n* = 3 days, 5 sample batches per day).

	Intra-Day			Inter-Day		
Theoretical Concentrations (ng/mL)	Concentration Measured (ng/mL)	Accuracy (%)	Precision (CV, %)	Concentration Measured (ng/mL)	Accuracy (%)	Precision (CV, %)
1	1.04 ± 0.04	103.76	3.81	1.00 ± 0.06	99.67	5.88
5	4.78 ± 0.22	95.69	4.50	4.75 ± 0.22	94.93	4.67
100	98.73 ± 1.84	98.73	1.86	98.65 ± 3.55	98.65	3.59
500	510.68 ± 13.82	102.14	2.71	500.40 ± 13.46	100.08	2.69

**Table 2:** Extraction recovery and matrix effect data for semaglutide.

	Extraction Recovery		Matrix Effect	
Concentration (ng/mL)	Recovery (%) ^a^	CV (%)	Matrix Effect (%) ^b^	CV (%)
5	93.05	2.47	102.22	9.84
100	107.95	1.63	96.34	2.96
500	99.31	1.68	104.12	3.23

ᵃ Extraction recovery was evaluated using independent sample batches (*n* = 3 sample batches). ᵇ Matrix effect, expressed as the IS-normalized matrix factor, was evaluated across six independent rat plasma lots, each prepared with K_2_ EDTA and analyzed in three sample batches; the reported values represent the mean and inter-lot CV of the six lots (*n* = 6 lots, 3 sample batches).

**Table 3:** Stability data for semaglutide (*n* = 3 sample batches).

	Bench-Top Stability (8 h at Room Temperature)			Long-Term Stability (5 Months at −80 °C)			Auto-Sampler Stability (24 h in the Auto-Sampler)			Freeze/Thaw Stability (3 Cycles)		
Theoretical Concentrations (ng/mL)	Mean (ng/mL)	% Nominal	CV, %	Mean (ng/mL)	% Nominal	CV, %	Mean (ng/mL)	% Nominal	CV, %	Mean (ng/mL)	% Nominal	CV, %
5	5.07 ± 0.13	101.39	2.53	4.78 ± 0.03	95.68	0.68	5.01 ± 0.40	100.10	8.05	4.65 ± 0.22	93.01	4.65
100	99.42 ± 2.15	99.42	2.16	100.29 ± 2.02	100.29	2.01	101.09 ± 1.04	101.09	1.03	101.56 ± 2.57	101.56	2.53
500	499.16 ± 7.30	99.83	1.46	489.38 ± 5.72	97.88	1.17	500.29 ± 2.69	100.06	0.54	478.41 ± 11.11	95.68	2.32

**Table 4:** Incurred sample reanalysis data for semaglutide.

	Incurred Sample Reanalysis		
Samples	Initial Concentration (ng/mL)	Reanalyzed Concentration (ng/mL)	Difference (%) ^†^
1	245.71	258.24	4.97
1	186.47	174.94	−6.38
2	214.39	219.64	2.42
2	177.00	184.09	3.93
3	213.46	208.88	−2.17
3	117.28	128.41	9.06
4	602.27	620.91	3.05
4	527.79	510.46	−3.34
5	588.35	559.55	−5.02
5	389.07	382.98	−1.58
6	372.64	396.50	6.20
6	293.37	271.93	−7.59
7	407.56	381.00	−6.74
7	330.34	333.12	0.84
8	447.13	457.29	2.25
8	316.99	312.54	−1.42

^†^ Expressed as [(repeat value − initial value)/mean value] × 100.

**Table 5:** Pharmacokinetic parameters of semaglutide. (mean ± SD, *n* = 8). C_max_, maximum peak plasma concentration; T_max_, time to reach C_max_; AUC_last_, area under the time–concentration curve from 0 h to the last measurable concentration; AUC_inf_, area under the time–concentration curve from 0 h to infinity; t_1/2_, terminal elimination half-life; CL/F, apparent clearance.

Parameter	Semaglutide
C_max_ (ng/mL)	419.07 ± 155.95
T_max_ (h)	7.25 ± 2.12
AUC_last_ (h•ng/mL)	8327.45 ± 3194.67
AUC_inf_ (h•ng/mL)	41,277.57 ± 30,079.18
t_1/2_ (h)	61.03 ± 30.88
CL/F (L/h)	0.002 ± 0.002

## Data Availability

The original contributions presented in this study are included in the article/Supplementary Materials. Further inquiries can be directed to the corresponding authors.

## Supplementary Materials

The following supporting information can be downloaded at: https://www.mdpi.com/article/10.3390/pharmaceutics18070770/s1, Figure S1: Representative calibration curve of semaglutide; Table S1: Gradient elution conditions and flow rates; Table S2: Back-calculated concentrations of the calibration standards for semaglutide (*n* = 3 independent runs over 3 days).

## Abbreviations

The following abbreviations are used in this manuscript:

LC–MS/MS	Liquid Chromatography–Tandem Mass Spectrometry
GLP-1	Glucagon-Like Peptide-1
LLOQ	Lower Limit of Quantification
MRM	Multiple Reaction Monitoring
