# Supplementary material for: Development and Validation of a Liquid Chromatography/Tandem Mass Spectrometry Method for the Quantification of the GLP-1 Analog Semaglutide in Rat Plasma, and Its Application in a Pharmacokinetic Study
Source: Pharmaceutics. 2026 Jun 24;18(7):770. doi: 10.3390/pharmaceutics18070770 (PMC13415238; doi:10.3390/pharmaceutics18070770)
Supplement: Supplementary file 1 [file pharmaceutics-18-00770-s001.zip › Supplementary material_R1.pdf]

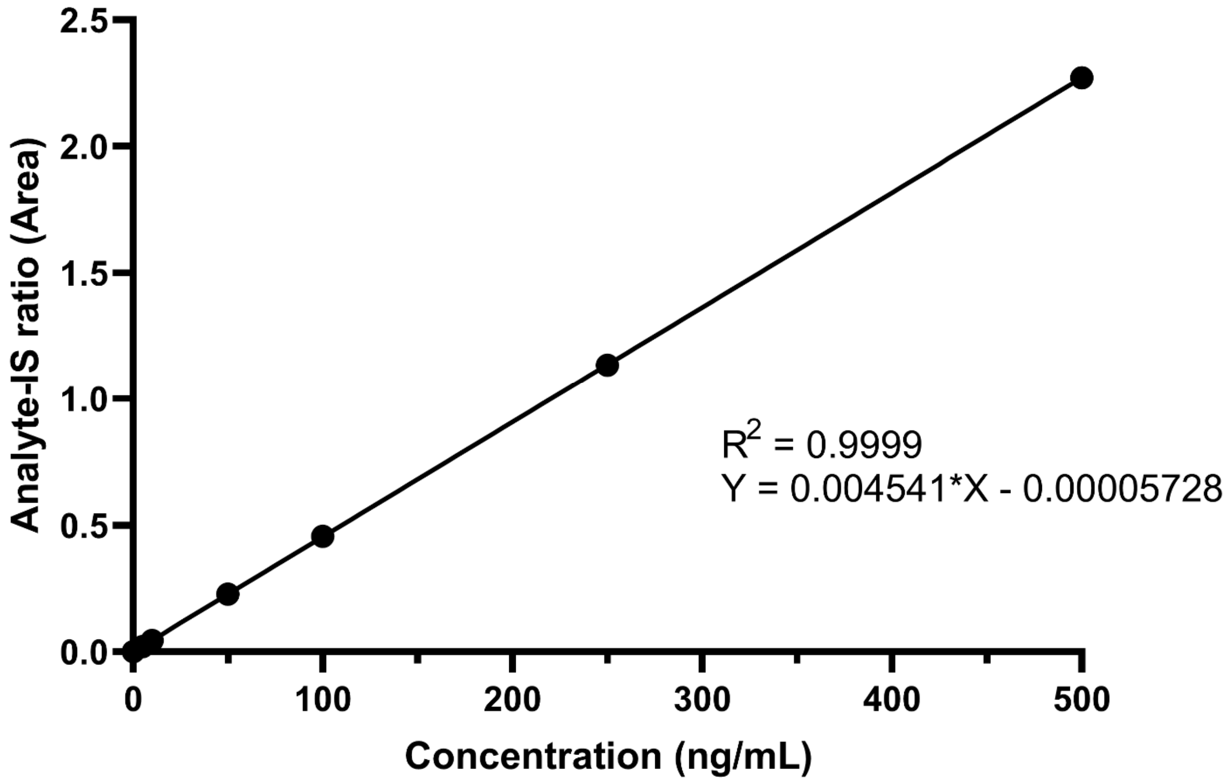


**Figure S1.** Representative calibration curve of semaglutide.

**Table S1.** Gradient elution conditions and flow rates.

| Step | Time (min) | 0.2% FA in ACN (%) | 1% FA in DW (%) | Flow rate (mL/min) |
|---|---|---|---|---|
| 1 | Initial | 38.0 | 62.0 | 0.28 |
| 2 | 1.0 | 38.0 | 62.0 | 0.28 |
| 3 | 2.4 | 60.0 | 40.0 | 0.28 |
| 4 | 5.0 | 60.0 | 40.0 | 0.28 |
| 5 | 5.0 | 38.0 | 62.0 | 0.28 |
| 6 | 9.0 | 38.0 | 62.0 | 0.28 |

**Table S2.** Back-calculated concentrations of the calibration standards for semaglutide (*n* = 3 independent runs over 3 days).

| Nominal Conc. (ng/mL) | Mean Back-Calculated Conc. (ng/mL) | Accuracy (%) | Precision (CV, %) |
|---|---|---|---|
| 1 (LLOQ) | 1.00 | 99.53 | 10.84 |
| 5 | 4.97 | 99.32 | 5.09 |
| 10 | 10.10 | 100.97 | 4.49 |
| 50 | 49.78 | 99.56 | 2.04 |
| 100 | 98.78 | 98.78 | 2.29 |
| 250 | 255.16 | 102.06 | 1.65 |
| 500 | 500.74 | 100.15 | 0.77 |

Accuracy is expressed as the percentage of the mean back-calculated concentration relative to the nominal concentration. Precision is expressed as the coefficient of variation (CV, %). All levels met the acceptance criteria of ±20% at the LLOQ and ±15% at all other concentrations.
